# Notes and News

**Published:** 1899-06-24

**Authors:** 


### Notes and News.

(Collected and Communicated.)

The Plague at Alexandria has not yet been entirely stamped out, two fresh cases being reported.

Mr. Passmore Edwards' Seaside Holiday Home for Children has been opened at Clacton-on-Sea, and he has made another donation of £1,000 to the institution.

Mr. E. Forster, M.A., has been appointed house governor and secretary of the Wolverhampton and Staffordshire General Hospital.

The *Graphic* this week contains an illustration of the laboratory at the recently-opened School for Tropical Diseases in Liverpool.

Professor Schafer, of University College, London, has been appointed Professor of Physiology at Edinburgh University, in place of the late Professor Rutherford.

Messrs. Macmillan and Co. announce for early publication a new volume on "Differential Diagnosis in Medicine," by Dr. Fred. J. Smith, of the London Hospital. It is chiefly intended for senior students.

An extraordinary discovery that snake serum is an effective cure for leprosy is reported to have been made by Dr. Dyar, of New York. It is also stated that the Government intend arranging for Dr. Dyar to establish a leper colony where the treatment can be put into practice.

One result of the Peace Conference at the Hague is the resolution to forward the observations of the provisions of the Geneva Conference in naval battles. It is proposed to paint hospital ships of a distinct colour, flying the Geneva flag, that combatants may easily recognise the ships conveying succour, and not interfere with their movements, and further that the ships shall be used equally by either side. The latter point may not be so easy of adoption, and it seems more suitable in every respect that each nationality joining in the combat should provide its own hospital ship. The probable difference of language between doctors and nurses and patients would serve as a drawback, to say nothing of the strain on the resources of one ship receiving consignments of invalids at the same time from both sides.

At a lecture delivered at Cirencester, Sir George Brown, C.B., formerly Professor of Veterinary Science at the Royal Agricultural College, expressed opinions of a more conservative nature in respect to the contagion of tuberculosis than are generally current at the present time. In respect to safeguarding the public against contagion through milk, he advocated boiling by the consumer rather than reliance on any precautions taken by the owners of cows and dairies, and he went as far as to intimate that he regarded the probability of contagion through animals at all as overestimated.

The eighteenth congress of the Sanitary Institute will be held in Southampton from August 29th to September 2nd. The president of the congress is Sir W. H. Preece, K.C.B., F.R.S., Pres.Inst.C.E.; and Bailie J. Dick, J.P., chairman of the Health Committee, Glasgow, will deliver the popular lecture. A health exhibition of apparatus and appliances relating to health and domestic use will be held as a practical illustration of the application and carrying out of the principles and methods discussed at the meetings. Excursions to places of interest in connection with sanitation will be arranged for those attending the congress. The local arrangements are in the hands of a committee, presided over by the Mayor of Southampton, with Mr. A. Wellesley Harris, M.R.C.S., D.P.H., Mr. W. B. G. Bennett, Asso.M.Inst.C.E., Mr. W. Matthews, M.Inst.C.E., and Mr. C. H. Russell, M.R.C.S., D.P.H., as hon. secretaries.

The Royal Orthopædic Hospital in Hanover Square is passing through troublous times. Its premises are old, and by all parties admitted to be unsuitable and unfit for their purpose. Its financial condition is also unsatisfactory, the income for some years having shown a declining tendency. And in addition to —no doubt largely on account of—this state of affairs discussions have lately arisen with regard to the management. It will be remembered that, as recorded in these columns at the time, at the annual meeeting in March—at which the then committee had announced its intention of bringing forward a scheme for selling the present premises and site, and erecting a new building elsewhere—seven members of the old committee were replaced by Mr. H. H. Marks, M.P., and his supporters, leaving Sir Walter Gilbey and his friends in a minority. Having thus been taken unawares and outvoted by a meeting packed for the purpose, principally, as subsequent examination showed, by persons who had been governors for less than four months, the members of the old committee called upon the president to convene a special court to make such alterations in the laws of the hospital as should render such tactics impossible in the future, feeling as they stated in a circular issued to the governors, "that the future of the hospital, and indeed of every similar charity, would be seriously imperilled if it were in the power of a small proportion of the governors, and those only very recently qualified as such, to reverse a seriously considered scheme without any notice whatever." In the result, as will be seen from our report of the proceedings, the old committee carried their proposals and then resigned in a body, Lord Wantage joining them in this step. The control of the institution, at any rate until the next election in 1900, is thus left entirely in the hands of Mr. Marks and his friends.

